# The Metastatic Process through the Eyes of Epigenetic Regulation: A Promising Horizon for Cancer Therapy

**DOI:** 10.3390/ijms232415446

**Published:** 2022-12-07

**Authors:** Bozena Smolkova, Julie Earl, Agapi Kataki

**Affiliations:** 1 Department of Molecular Oncology, Cancer Research Institute, Biomedical Research Center of the Slovak Academy of Sciences, Dubravska Cesta 9, 845-05 Bratislava, Slovakia; 2 Molecular Epidemiology and Predictive Tumor Markers Group, Medical Oncology Research Laboratory, Ramón y Cajal Health Research Institute (IRYCIS), Carretera Colmenar Km 9100, CIBERONC, 28034 Madrid, Spain; 3 1st Department of Propaedeutic Surgery, National and Kapodistrian University of Athens, Vasilissis Sofias 114, 11527 Athens, Greece

Genetic aberrations, including chromosomal rearrangements, loss or amplification of DNA, and point mutations, are major elements of cancer development. However, since epigenetic dysregulation was shown to be strongly related to human disease, especially cancer, the epigenetic component seems equally important. The term epigenetics was first introduced by Conrad Waddington in 1942 [1] in his effort to link the genotype with phenotype. It is currently used to describe the ensemble of several mechanisms that can reversibly modify gene expression profiles without altering the DNA sequence [2]. Epigenetic mechanisms are meant to cooperate with various other regulatory factors to secure time and tissue-specific regulation of gene expression in relation to developmental or environmental cues.

It was long assumed that tumors exhibit cell-to-cell variability, and recent technological advances have provided considerable evidence supporting gene expression and functional phenotypes heterogeneity within malignant tumors. Phenotypic plasticity seems to assist malignant cells in adapting to their environment in order to survive, grow and spread. Although most cancer cells leaving the primary tumor die in circulation, a small population, known as metastasis-initiating cells, survive and retain the ability to seed metastasis. These stem-like malignant progenitors adopt diverse phenotypic stages in response to intrinsic and external stromal signals driving their resistance to therapy and relapse [3]. Consequently, the vast majority of patients with recurrence or de novo metastases die within five years [4]. Acquired epigenetic and subsequent transcriptional changes have been shown as critical events in metastasis [5]. Excessive levels of enzymes that act as epigenetic modifiers have been reported as markers of aggressive cancers and associated with metastatic progression. Analysis of the mutation patterns and overall mutation burden in primary and metastatic cancers has been shown to be largely concordant [6,7]. Still, several recurrent metastasis-associated mutations were identified to be responsible for resistance to specific therapies. Recent studies have found that distinct subgroups of poor-prognosis tumors lack genetic alterations but are epigenetically regulated, confirming the critical role of epigenetic modifications and/or their modifiers to cancer progression [8].

Thus, this Special Issue, with one review and eight original research papers, has focused on deciphering genetic and epigenetic regulation of tumor progression and metastasis, providing novel insights into the mechanisms underlying processes associated with cancer cell plasticity and the development of metastatic disease.

The review of Ruscitto et al. focused on breast cancer, addressing genetic and phenotypic heterogeneity. Whole-genome sequencing of primary tumors and metastases revealed that breast cancer metastasis is a non-genetically selected trait resulting from transcriptional and metabolic adaptation to unfavorable microenvironmental conditions such as hypoxia, low nutrients, endoplasmic reticulum stress, or chemotherapy. However, the nature of the key players in the adaptive responses remains largely unknown [9]. Benhassine et al. analyzed the aberrant expression of serotonin receptor 2B (HTR2B), the most discriminant gene from the 12-gene expression signature, which can efficiently predict metastatic progression in uveal melanoma. The authors confirmed the presence of a STAT putative target site in the HTR2B promoter and showed the impact of IL-4 and IL-6 on HTR2B expression, thus providing evidence that HTR2B expression is modulated by STAT proteins [10].

One of the phenotypic plasticity processes relevant to the development of metastasis is the epithelial-to-mesenchymal transition (EMT), during which epithelial cells lose their polarity and cell–cell adhesion and invade the tumor stroma. Cells with EMT features are present at the invasion fronts of carcinomas [11]. Besides EMT, mesenchymal-to-epithelial transition (MET) endows the metastatic cells with traits needed to spread to distant organs. The dynamic shift between these two phenotypes indicates that the plasticity of EMT could be attributed to epigenetic regulation rather than to permanent genetic mutations [12]. Cancer cells with the mesenchymal state develop increased motility and invasive stem cell-like phenotype, including resistance to treatment [13]. The role of partial EMT phenotypes in prostate cancer progression was investigated by Kitz et al. Knockdown of Zeb1 resulted in partial EMT, inducing co-expression of EMT markers, a mixed epithelial/mesenchymal morphology, and increased invasion and migration. Treatment of knockdown cells with 5-azacytidine mitigated this aggressive phenotype. DNA methylation analysis using Illumina Methylation EPIC BeadChip revealed ten potential EMT targets, which can serve to identify patients who might benefit from 5-aza therapy [14]. Urbanova et al. used DNA methyltransferase inhibitor decitabine, which efficiently decreased DNA methylation by up to 53% and reactivated several silenced EMT-associated genes in four pancreatic ductal adenocarcinoma cell lines. These results confirmed the regulation of these genes by DNA methylation and uncovered possible new targets for epigenetic therapy. EMT plasticity suggests that epigenetic landscapes are implicated in the dynamic events underlying the EMT and might be responsible for tumor cell spread [15]. Dissemination of invasive ductal breast cancer cells through hematogenous or lymphomatous vessels was studied by Kalinkova et al. The authors interrogated the correlation between several miRNAs and EMT genes. As a result, they demonstrated the downregulation of miR-205-5p in CD45-depleted circulating tumor cell-positive tumor fraction and a negative correlation between miR-205-5p and *ZEB1* expression. These findings can potentially deliver markers for the metastatic behavior of disseminated tumor cells originating from invasive ductal carcinoma [16]. 

Cancer has long been regarded as a problem solely of cancer cells. However, the development and metastasis involve cross-talk between epithelial and stromal compartments mediated by paracrine signals and extracellular matrix [17]. Immune and non-immune cells control anti-metastatic defense or metastasis-supportive responses [18]. Cancer and stromal cell signaling influence one another, and this communication may co-evolve during the course of tumor progression. The maturity stage of mesenchymal stromal cells involved in tissue regeneration, immune modulation, and secretion of angiogenic molecules, cytokines, and paracrine factors was studied by Manocha et al. [19]. The authors demonstrated that in fat tissue, CD146-expressing cells might represent a more mature pericyte subpopulation having higher efficacy in controlling and stimulating vascular regeneration and stabilization than their CD146-negative counterparts. 

Through their cargo, consisting of various molecules, including DNA, miRNA, siRNA, and proteins, extracellular vesicles are important mediators of cell-to-cell communication. In recent years, many studies have focused on exosomes and their role in cancer progression and metastasis [20]. The potential of carboxypeptidase E (CPE) as an exosomal bioactive molecule driving the growth and invasion of low-metastatic hepatocellular carcinoma cells was studied by Hareendran et al. [21]. The authors showed that CPE is a key player in the exosome-based delivery of CPE-shRNA, which offers a potential treatment for hepatocellular carcinoma and utility as a liquid biopsy tool. 

In many cancers, surgical resection of the primary tumor is followed by a period without evidence of disease followed by aggressive metastatic growth. The study by Lin et al. identified gene expression signatures able to predict 100% of brain-metastasizing lung adenocarcinoma tumors with a 91% specificity, thus facilitating the detection of patients at the highest risk of brain metastasis by analyzing primary tumors [22]. These findings demonstrate that cancer-glia/neuron interaction may play a fundamental role in developing lung cancer brain metastasis.

As mentioned earlier, during tumorigenesis, cancer cells face a variety of intrinsic and extrinsic stresses, forcing the activation of several mechanisms, including autophagy which is often characterized as a double-edged sword, and its role is still under investigation [23]. Zaarour et al. explained why waterpipe smokers with lung adenocarcinoma and an increase in autophagy-activating genes, higher mutation burden, and CD8+ T-cell levels respond better to immunotherapy, despite a lack of differences in immune checkpoint gene PD-1, PD-L1, PD-L2 and CTLA-4 expression [24].

We believe that deciphering the role of genetic and epigenetic changes and their regulatory mechanisms in cancer progression will be crucial for the further molecular understanding of the metastatic process. Epigenetic therapies targeting epigenetic regulators could have a major clinical impact on the development of next-generation drugs, especially when combined with new preclinical patient-derived preclinical models. In addition, given the potential of novel generations of epigenetic inhibitors, the characterization of specific epigenetic subtypes may lead to better patient stratification. Targeting epigenetic modifiers and modifications represents an innovative strategy for treating disease and delaying or preventing resistance to other anticancer therapies in solid tumors [25].
